# Oligometastatic disease in esophagogastric cancer: an update of recommendations on definition, diagnosis, and treatment

**DOI:** 10.1016/j.esmogo.2023.08.008

**Published:** 2023-11-27

**Authors:** T.E. Kroese, S.F.C. Bronzwaer, R. van Hillegersberg, P.S.N. van Rossum, H.W.M. van Laarhoven

**Affiliations:** 1 Department of Radiation Oncology, University Hospital Zurich, Zurich University, Zurich, Switzerland; 2 Department of Surgery, University Medical Center Utrecht, Utrecht University, Utrecht; 3 Department of Radiation Oncology, Amsterdam UMC, Location VUmc, Amsterdam; 4 Department of Medical Oncology, Amsterdam UMC, Location University of Amsterdam, Amsterdam; 5 Cancer Center Amsterdam, Cancer Treatment and Quality of Life, Amsterdam, The Netherlands

**Keywords:** esophageal cancer, gastric cancer, oligometastasis, stereotactic body radiotherapy, metastasectomy, Delphi consensus

## Abstract

Oligometastatic cancer is characterized by the presence of a restricted number of metastatic lesions extending beyond the primary tumor. Until recently, there was a significant lack of agreement concerning the precise definition and optimal treatment strategies for oligometastatic cancer in the context of esophageal or gastric malignancies. Here we provide an overview of the OligoMetastatic Esophagogastric Cancer (OMEC) project which was initiated to develop a multidisciplinary European consensus statement for the definition, diagnosis, and treatment of oligometastatic esophagogastric cancer. Additionally, we provide an updated systematic review of published and ongoing clinical studies on local metastasis-directed treatment of oligometastatic esophagogastric cancer.

## Highlights

• A multidisciplinary consensus definition for esophagogastric OMD was developed.
• One organ with ≤3 metastases is considered OMD.
• One extra-regional lymph node station is considered OMD.
• Patients with OMD undergo systemic therapy followed by restaging.
• Patients without progression at ^18^F-FDG PET/CT restaging undergo local treatment.

## Graphical abstract

**Figure:**
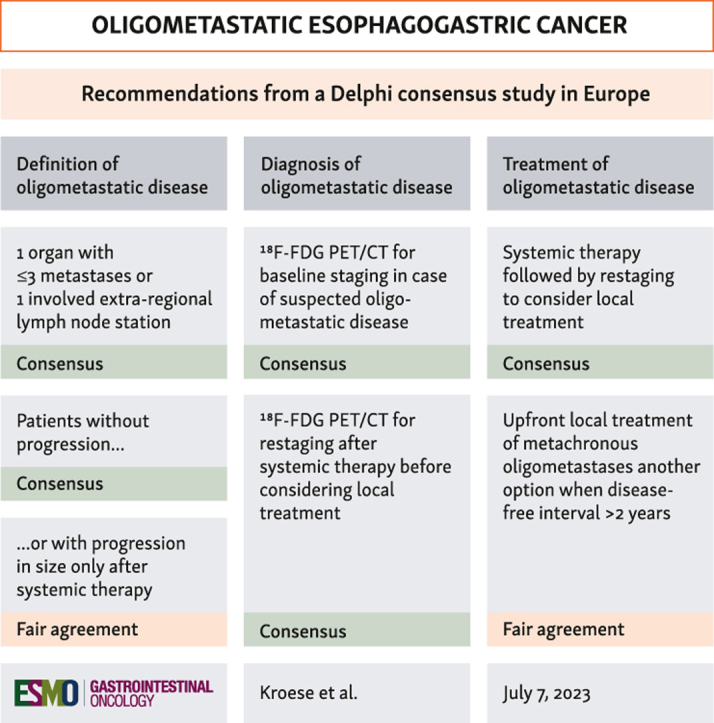

## Introduction

Oligometastatic esophagogastric cancer refers to the presence of a limited number of metastatic lesions beyond the primary tumor in the esophagus or stomach.^1^ Oligometastatic disease is believed to encompass unique biological and clinical features, challenging the conventional dichotomy of cancer as either localized and potentially curable or systemic and non-curable. This concept recognizes the existence of an intermediate state where cancer demonstrates limited metastatic spread, suggesting the possibility of curative treatment approaches if metastases are amenable to local therapies, such as metastasectomy or stereotactic body radiotherapy (SBRT).^2^, ^3^, ^4^ Commonly accepted criteria for oligometastatic disease involve the presence of up to three to five metastatic lesions.^2^^,^^3^ The precise number of metastatic lesions that define oligometastatic disease in the context of esophagogastric cancer is, however, subject to ongoing debate.

Oligometastatic esophagogastric cancer is a significant health care burden worldwide. A multicenter retrospective cohort study of patients with metastatic esophagogastric cancer showed that the incidence of oligometastatic disease (defined in that study as five or fewer distant metastases) was 24%.^5^ Another population-based cohort study of patients with gastric cancer with synchronous metastatic disease limited to the liver showed that the incidence of oligometastatic disease was 26%.^6^ Consequently, it becomes imperative to establish tailored treatment guidelines for these patients, which up until now have been lacking.

The OligoMetastasis in Esophagogastric Cancer (OMEC) project was initiated to develop a multidisciplinary European consensus statement for the definition, diagnosis, and treatment of oligometastatic esophagogastric cancer.^7^ In this review we will report the findings of the OMEC project thus far^8^, ^9^, ^10^ and additionally provide an updated systematic search of ongoing and published phase II-III clinical trials on oligometastatic esophagogastric cancer on clinicaltrials.gov or PubMed up to 29 June 2023.

### Defining the concept of oligometastatic esophagogastric cancer

**Figure 1:**
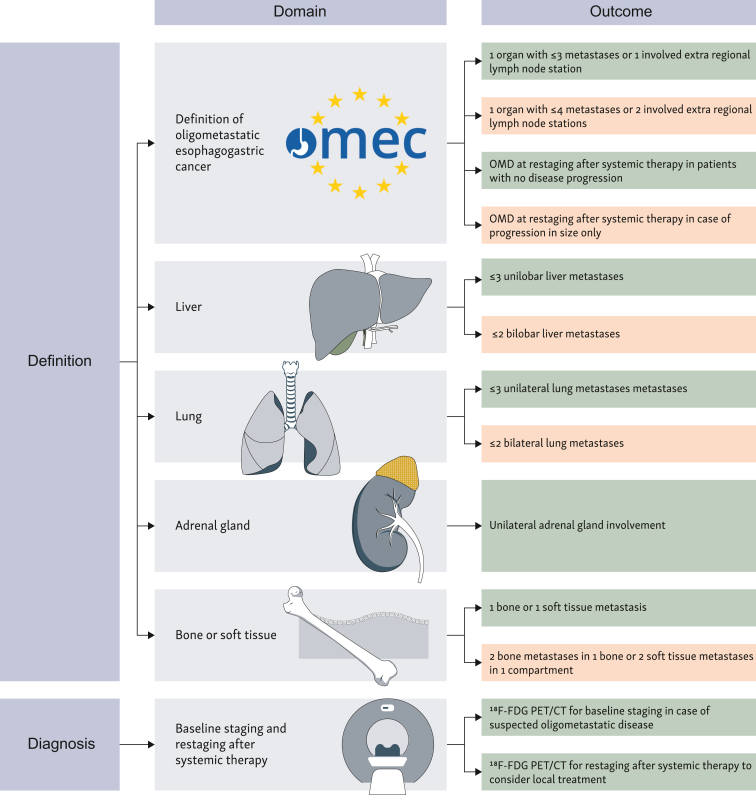
**Definition and diagnosis of oligometastatic esophagogastric cancer according to the OMEC project.** Green boxes indicating consensus (i.e. ≥75 agreement), orange boxes indicating fair agreement (50%-75% agreement). ^18^F-FDG PET–CT, [^18^F]2-fluoro-2-deoxy-D-glucose positron emission tomography–computed tomography; OMD, oligometastatic disease; OMEC, OligoMetastatic Esophagogastric Cancer.

Published and ongoing studies use various definitions of oligometastatic disease. Most studies (50%) consider a maximum of one involved organ (with or without the retroperitoneal lymph nodes) with three or fewer metastases as oligometastatic disease (Table 1). In the OMEC project, we achieved consensus (i.e. ≥75% agreement) on the definition of oligometastatic disease being three or fewer distant metastases in a maximum of one organ or one involved extraregional lymph node station.^10^ In addition, we achieved agreement on the definition of oligometastatic disease after systemic therapy. Patients without progression of oligometastatic lesions after systemic therapy should be considered to still have oligometastatic disease (consensus).^10^ Also, patients with progression in only the size of existing lesions after systemic therapy, but not in the number of lesions, could be considered to still have oligometastatic disease (fair agreement).^10^ Figure 1 provides a schematic overview of the recommendations on the definition and diagnosis of oligometastatic esophagogastric cancer according to the OMEC project.

**Table 1:** Overview of completed and ongoing trials in patients with oligometastatic esophagogastric cancer

	Author/sponsor name or clinicaltrials.gov ID	Primary tumor	Country	Study type	Maximum number of organs	Maximum number of metastases	Type of OMD	Staging	Treatment	Median overall survival
Completed	Zhao et al., 2023^11^	Esophageal SCC	China	Phase II NR	ns	5	Synchronous/metachronous	ns	IO + ChT + SBRT	12.8 Months
	Cui et al., 2023^12^	Gastric AC	China	Phase II NR	1	Organ-specific	Synchronous	CT or laparoscopy	ChT + surgery + ChT	Not reached
	Liu et al., 2020^13^	Esophageal SCC	Chins	Phase II NR	ns	3	Metachronous	CT or ^18^F-FDG PET	SBRT +/− ChT	24.6 Months
	Al-Batran et al., 2017^14^	Gastric AC or EGJ AC	Germany	Phase II NR	1 + RPLN	Organ-specific	Synchronous	CT/MRI or ^18^F-FDG PET	ChT + surgery	31.3 Months
Ongoing	NCT04510064 (Fudan University)^15^	Gastric AC or EGJ AC	China	Phase II NR	1	Organ-specific	Synchronous	CT or MRI	IO + ChT + surgery	NA
	NCT04248452 (ECOG-ACRIN Cancer Research Group)^16^	Esophageal AC and gastric	USA	Phase III R	ns	3	Synchronous	CT or MRI	ChT + SBRT versus ChT	NA
	NCT03904927 (Fudan University)^17^	Esophageal SCC	China	Phase II R	2	4	Synchronous/metachronous	CT	ChT + SBRT/Surgery versus ChT	NA
	NCT03161522 (M.D. Anderson Cancer Cancer)^18^	Esophageal AC	USA	Phase II NR	1	3	Synchronous	^18^F-FDG PET/CT	ChT + SBRT/surgery	NA
	NCT03399253 (Sun Yat-sen University)^19^	Gastric AC	China	Phase II-III R	2	Organ-specific	Synchronous	CT	ChT + surgery versus ChT	NA
	NCT02578368 ‘FLOT5’ (Krankenhaus Nordwest)^20^	Gastric AC or EGJ AC	Germany	Phase III R	1 + RPLN	Organ-specific	Synchronous	CT/MRI or ^18^F-FDG PET	ChT + surgery versus ChT	NA
	NCT04512417 (Zhejiang Cancer Hospital)^21^	Esophageal SCC or AC	China	Phase II R	ns	4	Synchronous/metachronous	ns	IO + ChT + SBRT versus IO + ChT	NA
	NCT03042169 ‘Surgigast’ (University Hospital Lille)^22^	Gastric AC or EGJ AC	France	Phase III R	1 + RPLN	Organ-specific	Synchronous	CT/MRI or ^18^F-FDG PET	ChT + surgery versus ChT	NA

^18^F-FDG–PET, fluorodeoxyglucose position emission tomography; AC, adenocarcinoma; ChT, chemotherapy; CT, computed tomography; EGJ, esophagogastric junction; IO, immune-oncology; MRI, magnetic resonance imaging; NA, not applicable; NR, non-randomized; ns, not specified; OMD, oligometastatic disease; R, randomized; RPLN, retroperitoneal lymph nodes; SBRT, stereotactic body radiotherapy; SCC, squamous cell carcinoma.

### Diagnostic modalities for oligometastatic disease

Advances in imaging technology have made it possible to detect small metastases. This has led to a better distinction between oligometastatic and polymetastatic disease, allowing patients with more widespread disease to avoid unnecessary local treatment. Most importantly, [^18^F]2-fluoro-2-deoxy-d-glucose positron emission tomography with integrated computed tomography (^18^F-FDG PET–CT) has demonstrated improved identification of patients with oligometastatic burden who could potentially benefit from radical treatment strategies.^23^ Despite these advancements, these imaging modalities have limitations in detecting microscopic metastatic lesions or differentiating between active disease and inflammation.^24^ This (and limited availability in some centers) might explain why published and ongoing studies are using various diagnostic modalities to stage oligometastatic disease. Fortunately, most recent studies in patients with oligometastatic esophagogastric cancer used radiological imaging techniques such ^18^F-FDG PET–CT and/or magnetic resonance imaging (MRI) to evaluate the extent of metastatic disease.^14^, ^18^, ^20^, ^22^, ^25^ In the OMEC project, it is recommended to use ^18^F-FDG PET–CT for baseline staging in case of suspected oligometastatic disease in order to exclude patients with polymetastatic disease (consensus).^10^ In addition, it is recommended to use ^18^F-FDG PET–CT for restaging after systemic therapy to consider local treatment (consensus).^10^

### Treatment of oligometastatic disease

**Figure 2:**
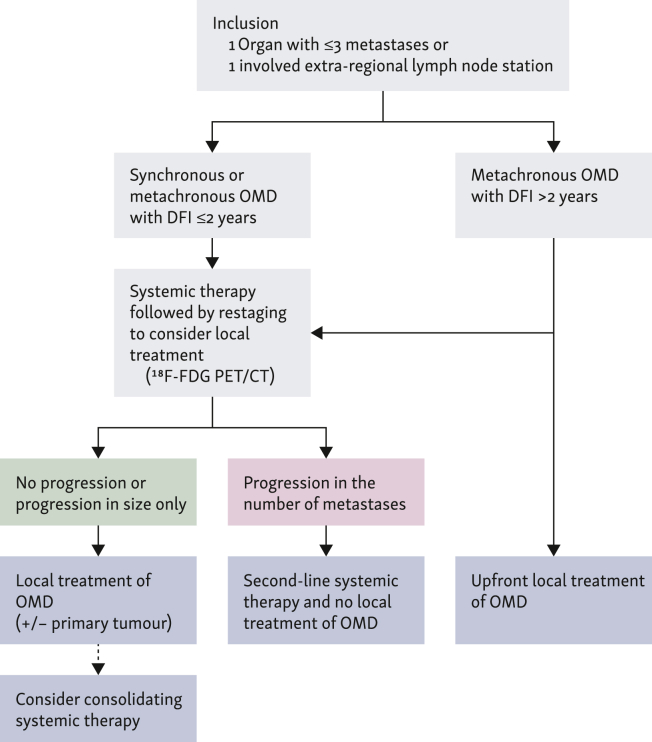
**A suggested treatment algorithm for oligometastatic esophagogastric cancer according to the OMEC project.** ^18^F-FDG PET–CT, [^18^F]2-fluoro-2-deoxy-D-glucose positron emission tomography–computed tomography; DFI, disease-free interval; OMD, oligometastatic disease; OMEC, OligoMetastatic Esophagogastric Cancer.

Published and ongoing studies are using various treatment options for treatment of oligometastatic disease. For treatment of metastases, studies including patients with gastric adenocarcinoma or synchronous oligometastatic disease are predominantly using surgery (i.e. metastasectomy),^14^^,^^20^^,^^22^ whereas studies including patients with esophageal squamous cell cancer or metachronous oligometastatic disease are predominantly using SBRT.^11^^,^^13^ In the OMEC project, recommended treatment of patients with synchronous or metachronous oligometastatic disease with a disease-free interval of ≤2 years is systemic therapy followed by restaging to consider local treatment (consensus).^10^ For patients with metachronous oligometastatic disease with a disease-free interval >2 years, the recommended treatment could either consist of upfront local treatment of oligometastatic disease or systemic therapy followed by restaging to consider local treatment (fair agreement).^10^ The type of local treatment (e.g. metastasectomy or SBRT) is to be decided by the local multidisciplinary team based on individual patient and tumor characteristics and available expertise per center.^10^ Figure 2 provides a suggested treatment algorithm for oligometastatic esophagogastric cancer according to the OMEC project.

### Overall survival after local treatment of oligometastatic disease

Four prospective phase II non-randomized trials on local treatment of oligometastatic esophagogastric cancer have been completed (Table 1).^11^, ^12^, ^13^, ^14^ Two Chinese single-arm trials including patients with esophageal squamous cell cancer investigated survival outcomes after SBRT in combination with camrelizumab and second-line irinotecan chemotherapy,^11^ or SBRT with or without physician-choice chemotherapy.^13^ In addition, a Chinese multicenter prospective single-arm phase II trial including patients with gastric adenocarcinoma and synchronous oligometastatic disease investigated survival outcomes after surgical resection of the primary tumor and metastases in patients responding to docetaxel, oxaliplatin, and S-1 chemotherapy.^12^ In these three studies, median overall survival was 12.8 months,^11^ 24.6 months,^13^ or median overall survival was not reached after a median follow-up time of 30.0 months,^12^ respectively. A German phase II non-randomized trial including patients with synchronous gastric or gastroesophageal junction cancer investigated survival outcomes after fluorouracil, leucovorin, oxaliplatin, and docetaxel (FLOT) chemotherapy and subsequent resection of the primary tumor and metastases in patients with response to FLOT chemotherapy.^14^ Median overall survival in patients with response to FLOT chemotherapy and resection of the primary tumor and metastases was 31.3 months whereas overall survival in patients with progression who did not respond to FLOT chemotherapy and did not undergo resection was 15.9 months.^14^ These studies including highly selected patients showed favorable overall survival compared with historical cohorts of patients with metastatic esophagogastric cancer undergoing systemic therapy alone (generally 4-12 months) ‘suggesting’ a potential benefit of additional local metastasis-directed treatment.^26^^,^^27^ Due to the non-randomized study designs, however, the benefit of local treatment of oligometastatic disease over systemic therapy alone cannot be concluded.

Currently, nine ongoing phase II-III prospective clinical trials are investigating local treatment (with or without systemic therapy) in patients with oligometastatic esophagogastric cancer (Table 1). The majority of these ongoing trials are from China (55%), and include patients with synchronous oligometastatic disease (78%). Three ongoing studies are prospective phase III randomized trials.^16^^,^^20^^,^^22^ These trials investigate the benefit of systemic therapy plus local treatment of oligometastatic disease over systemic therapy alone for oligometastatic disease.^16^^,^^20^^,^^22^ Two of these studies are from Europe (the so-called ‘Renaissance/FLOT-5’ trial and the ‘Surgigast’ trial).^20^^,^^22^ Both studies encompass patients with gastric or gastroesophageal junction adenocarcinoma and synchronous oligometastatic disease.^20^^,^^22^ These studies aim to evaluate the potential improvement in overall survival by performing resection of the primary tumor and metastases in patients who respond to FLOT chemotherapy, in comparison to those who receive chemotherapy alone and continue their treatment course.^20^^,^^22^ Furthermore, a study conducted by the Eastern Cooperative Oncology Group and the American College of Radiology Imaging Network (ECOG-ACRIN) Cancer Research Group in the United States is investigating the potential overall survival advantage of SBRT in patients who demonstrate a positive response to chemotherapy, in contrast to continuing with chemotherapy alone.^16^

## Discussion

Oligometastatic esophagogastric cancer represents a distinct entity within the realm of metastatic disease, challenging the traditional notion of incurability. The paradigm shift towards local treatment in addition to systemic therapy offers a potential curative option for selected patients. The OMEC project has led to a multidisciplinary European consensus for the definition, diagnosis, and treatment of oligometastatic esophagogastric cancer that paves the way for improved survival outcomes.^10^ The collaborative design with a multidisciplinary approach, involving radiation oncologists, medical oncologists, and surgical oncologists who are recognized as experts in the field ensured a well-rounded perspective and expertise in managing this complex disease.^7^ The project’s predominantly European focus means, however, that the majority of experts involved have primarily dealt with esophagogastric adenocarcinoma, whereas squamous cell carcinoma is more commonly found in Asian countries. As a result, the project’s recommendations may not comprehensively represent the perspectives of experts outside of Europe, especially in the context of squamous cell carcinoma.

Numerous complexities persist in the management of oligometastatic esophagogastric cancer. Foremost, patient management is hindered by the limited availability of substantial evidence to guide clinical decision-making. Consequently, health care professionals have to rely on non-randomized phase II data,^11^^,^^12^,^14^^,^^25^ clinical case discussions,^9^ and expert opinions.^10^ Furthermore, the number of ongoing phase III studies in this field remains scarce.^16^^,^^20^^,^^22^

Accurate patient selection—i.e. distinguishing those who may benefit from local therapies from those who have more widespread metastatic disease and may require systemic therapies alone—is key in the management of oligometastatic disease. Due to the remaining inherent limitations in the sensitivity of current radiological techniques, there is a risk of potential misclassification of patients, leading to an incorrect diagnosis of oligometastatic disease when patients may actually have a higher metastatic burden. Certain molecular markers may show promise in providing enhanced sensitivity for the diagnosis of oligometastatic disease, either independently or in combination with radiological imaging techniques. For example, circulating tumor DNA (ctDNA) has emerged as a promising prognostic and predictive factor in both localized and metastatic esophagogastric cancer. Two cohort studies including 45 patients with esophageal cancer^28^ and 40 patients with gastroesophageal adenocarcinoma,^29^ respectively, who underwent ctDNA assessment before treatment showed that the presence of ctDNA after treatment (i.e. molecular relapse) preceded clinical/radiologic relapse. Therefore, ctDNA assessment could be used for minimal residual disease detection to identify patients at high risk for tumor progression after treatment.^28^^,^^29^ As such, ctDNA to stratify patients in high- and low-risk groups might prove useful to guide intensification or de-escalation of subsequent treatment such as consolidating systemic therapy after local treatment of oligometastatic disease.

Moreover, the utilization of ctDNA for response assessment during systemic therapy has proven valuable. It has been observed that patients with gastric cancer who still exhibit residual ctDNA following systemic therapy experience worse overall survival.^30^ An ongoing randomized study is currently incorporating the concept of ctDNA response assessment in the management of patients with synchronous or metachronous oligometastatic adenocarcinoma of the foregut.^31^ Patients in this study will first undergo systemic therapy and patients who have shown no progression after induction chemotherapy and have undetectable ctDNA levels will subsequently be randomized in a 1 : 1 ratio to either sequential curative (local) intervention followed by maintenance chemotherapy or routine continuation of chemotherapy.^31^ The primary endpoint of this study is progression-free survival, and a total of 48 patients will be enrolled.^31^ This is the first phase II randomized study evaluating the efficacy and safety of local treatment of oligometastatic disease in ctDNA-negative patients.^31^

Additionally, research is needed to determine what the ideal timing is for the administration of local therapies and systemic therapies in relation to each other. Should systemic therapies be applied only before or also after local treatment and what should the duration and type of systemic therapy be? Currently, the duration of chemotherapy in ongoing phase III studies including patients with oligometastatic esophagogastric varies widely.^16^^,^^20^^,^^22^ In the Renaissance/FLOT-5 study, patients first undergo four cycles of FLOT and patients with response are subsequently randomized to four to eight additional cycles of FLOT plus surgery of the primary tumor and metastases or continuation of four to eight cycles of FLOT alone.^20^ Patients in the phase III trial by the ECOG-ACRIN research group will first undergo six cycles of capecitabine and oxaliplatin (CapOx) or four cycles of FLOT and patients with response will be randomized to SBRT plus 2 years of CapOx/FLOT or 2 years of CapOx/FLOT alone.^16^

Of note, these phase III studies are not incorporating immunotherapy in the treatment algorithm.^16^^,^^20^ The CheckMate 577 trial has shown improved disease-free survival with adjuvant nivolumab compared with placebo in patients with esophageal cancer with an incomplete pathological response to neoadjuvant chemoradiotherapy and surgery.^32^ This was in contrast to the recently completed ATTRACTION-5 trial, which failed to show improved relapse-free survival after adjuvant nivolumab plus chemotherapy compared with adjuvant chemotherapy alone in patients with pathological stage II-III gastric cancer who had undergone gastrectomy.^33^ In contrast, preliminary non-peer reviewed results of studies incorporating checkpoint inhibitors in the perioperative setting show improved pathological response data, although these may not translate in survival benefit.^34^, ^35^, ^36^, ^37^ Altogether, these results suggest a potential role for immunotherapy in combination with chemotherapy and local treatment of oligometastatic disease, but the precise timing of these treatments is still uncertain.

Finally, the implementation of the management of oligometastatic disease may encounter obstacles due to increasing costs. For example, the use of advanced diagnostic techniques, such as molecular profiling and imaging modalities, may be necessary to accurately identify and characterize oligometastatic lesions, while follow-up and surveillance of patients with oligometastatic disease may require frequent imaging scans, laboratory tests, and clinical visits. These ongoing monitoring efforts can contribute to increased health care costs over time and may pose a challenge for the health care system, particularly in resource-limited settings. Finally, the implementation of comprehensive care models, involving multidisciplinary teams and specialized clinics, including surgery, radiation therapy, and systemic therapies are needed to provide optimal management for patients with oligometastatic disease. These models often involve additional resources and infrastructure, which can add to the overall cost burden and may not be accessible in certain countries or all parts of a country, thereby limiting their availability to all patients.

In conclusion, the development of recommendations for the definition, diagnosis, and treatment of oligometastatic esophagogastric cancer based on the best available evidence is crucial. It is of utmost importance to substantiate and validate these recommendations through rigorous clinical trials. The eagerly anticipated results from ongoing clinical trials will play a pivotal role in guiding evidence-based medicine and improving patient care in this field.
